# Effectiveness of Online and Remote Interventions for Mental Health in Children, Adolescents, and Young Adults After the Onset of the COVID-19 Pandemic: Systematic Review and Meta-Analysis

**DOI:** 10.2196/46637

**Published:** 2024-02-05

**Authors:** Linda Fischer-Grote, Vera Fössing, Martin Aigner, Elisabeth Fehrmann, Markus Boeckle

**Affiliations:** 1 Department of Psychology and Psychodynamics Karl Landsteiner University of Health Sciences Krems Austria; 2 Department of Clinical Psychology and Psychotherapy University Hospital Krems Krems Austria; 3 Research Centre Transitional Psychiatry Karl Landsteiner University of Health Sciences Krems Austria; 4 Department of Psychiatry for Adults University Hospital Tulln Tulln Austria

**Keywords:** COVID-19 pandemic, online/digital mental health intervention, e-mental health, anxiety, social functioning, depression, well-being, psychological distress, eating disorder, COVID-19 symptoms

## Abstract

**Background:**

The prevalence of mental illness increased in children, adolescents, and young adults during the COVID-19 pandemic, while at the same time, access to treatment facilities has been restricted, resulting in a need for the quick implementation of remote or online interventions.

**Objective:**

This study aimed to give an overview of randomized controlled studies examining remote or online interventions for mental health in children, adolescents, and young adults and to explore the overall effectiveness of these interventions regarding different symptoms.

**Methods:**

A systematic literature search was conducted according to PRISMA (Preferred Reporting Items for Systematic Reviews and Meta-Analysis) guidelines using PubMed, PsycInfo, Psyndex, Embase, and Google Scholar. A meta-analysis was conducted using a random effects model to calculate overall effect sizes for interventions using standardized mean differences (SMDs) for postintervention scores.

**Results:**

We identified 17 articles with 8732 participants in the final sample, and 13 were included in the quantitative analysis. The studies examined different digital interventions for several outcomes, showing better outcomes than the control in some studies. Meta-analyses revealed significant medium overall effects for anxiety (SMD=0.44, 95% CI 0.20 to 0.67) and social functioning (SMD=0.42, 95% CI –0.68 to –0.17) and a large significant effect for depression (SMD=1.31, 95% CI 0.34 to 2.95). In contrast, no significant overall treatment effects for well-being, psychological distress, disordered eating, and COVID-19–related symptoms were found.

**Conclusions:**

The qualitative and quantitative analyses of the included studies show promising results regarding the effectiveness of online interventions, especially for symptoms of anxiety and depression and for training of social functioning. However, the effectiveness needs to be further investigated for other groups of symptoms in the future. All in all, more research with high-quality studies is required.

## Introduction

The high prevalence of psychological disorders in children and adolescents is well known, has been reported for a long time [1-4], and was estimated in 2015 to be 13.4% worldwide [4]. Psychological disorders in these age groups often show long-term impacts on adult life as well [2,5]. Childhood and adolescence are relevant periods for learning and brain maturing, possibly resulting in either a positive or negative impact [6]. Due to these developmental aspects, adolescents have, for example, been found to be especially vulnerable to addiction and addictive behavior [7].

Because of the COVID-19 pandemic and all the accompanying characteristics, prevalence rates of mental health issues have increased in the general population [8], adolescents [9], and young adults, who are among the groups most at risk of suffering from a COVID-19–related decrease in mental health [10-15]. A systematic review reported a lockdown-associated increase in anxiety and depressive symptoms in children and adolescents and an increase in sleep disorders; as risk factors, a priori mental illness and high media exposure were identified [16]. Increased stress levels are associated with respective containment measures [15].

Earlier research spanning from 1946 until 2020 showed an increased risk of depression and anxiety in children and adolescents due to loneliness and isolation [17]. This is an important aspect the current pandemic brought about in many countries [9,17] due to lockdowns and homeschooling, possibly impacting adolescents especially, as emotional support by peers is highly relevant at this age [9]. School closures resulted in a change in daily routines, which is particularly important for young people with mental health problems. Additionally, social isolation poses a risk factor for domestic violence, and an increase in worries related to the future, like school success, university access, and employment chances, has been noted [18].

However, the negative impact of the pandemic consists not only of an increase in mental health issues but also a significant impediment to the accessibility of treatment options, among other aspects, due to the need for social distancing [8,19]. Even before the pandemic, some groups of patients, like migrants [20], different groups of minorities [21], and people in remote areas [22], were difficult to reach through mental health programs. Prior to the pandemic, fewer than 50% of adolescents with depression used adequate services [23,24].

The sudden onset and accompanying restrictions of the pandemic made it even more necessary to increase the offers of online therapy to maintain the treatment of patients with mental health issues. These offers led to a sudden increase in therapists using online interventions [8,25-27], thereby seemingly decreasing perceived barriers by psychotherapists to use online or remote treatment options [8,26]. Nevertheless, the sudden switch also resulted in insecurities and the need for further guidance for therapists [27].

New media and online interventions have been developed and studied for years now [8], including in the context of children and adolescents with psychosomatic illnesses [28], with some studies even finding advantages of virtual therapy compared with face-to-face treatments [29] or at least similar outcomes [30]. Generally, reasonable user satisfaction and feasibility of interventions have been found [30], and studies show that therapeutic alliances can also be reached during videoconferencing, with clients rating bond and presence as equal to face-to-face settings [22]. Online help-seeking seems related to increased anonymity, accessibility, and inclusivity [31], and social media shows benefits for offering mental health care [32]. Applications developed to enhance mental health in children and adolescents show good acceptability [33]. Co-designed eHealth for adolescents appears to be associated with a more engaging and satisfying user experience [34-37].

Still, more research on effectiveness is needed [33], especially considering the sudden switch to online therapies due to COVID-19. Some reviews have been conducted regarding the effectiveness of online interventions for mental health related to the COVID-19 pandemic [38-41]; a review by Bonardi et al [38] focused on randomized controlled trials (RCTs) explicitly designed to address mental health issues related to COVID-19 and found some with promising effects but none for children or adolescents that met the inclusion criteria. Regarding web-based exercise interventions for depressive symptoms and anxiety, a review found no clear recommendations [39], while Valentine et al [40] found telehealth services for neurodevelopmental disorders to be primarily equal to control groups and focused on studies conducted before the onset of the COVID-19 pandemic. Yunus et al [41] found efficacy of digitalized interventions for depression in pregnant women and included studies from before the pandemic.

Nevertheless, it seems of high relevance to identify studies of interventions for mental health conducted after the onset of the pandemic with children, adolescents, and young adults, as persons of these age groups are at a higher risk of being negatively impacted by the pandemic in the long term. Whereas children, adolescents, and young adults can be considered “digital natives” [42], which might make them especially receptive to online interventions, younger individuals also seem to be especially vulnerable to negative aspects of digital media usage (eg, problematic smartphone use) [43]. Not only is it necessary to identify RCTs studying these aspects but one should also take into consideration the specific type of control condition that is used since different types of controls lead to different strengths of studies and especially in mobile health interventions, the combination of a variety of features might account for the resulting effects [44].

Thus, this systematic review and meta-analysis aimed to give a concise overview of studies examining the effectiveness of online or remotely delivered interventions or interventions delivered through digital media since the onset of the COVID-19 pandemic for specific mental health issues in children, adolescents, and young adults.

## Methods

### Search Strategy

To identify papers published since early 2020 (after the initial onset of the COVID-19 pandemic) until June 2023, a literature search based on the PRISMA (Preferred Reporting Items for Systematic Reviews and Meta-Analysis) framework [45] was conducted in PubMed, PsycINFO, Psyndex, Embase*,* and Google Scholar. The detailed search parameters are depicted in Textbox 1. The reference search strategy was applied to locate additional relevant studies, and Google Scholar alerts were enabled. Multimedia Appendix 1 shows the PRISMA checklist, while Multimedia Appendix 2 shows the search strategy in more detail.

**Search parameters used in the literature search.**

Databases

- PubMed
- PsycINFO
- Psyndex
- Embase

Search parameters

- (((depression) OR (anxiety) OR (mental health) OR (eating disorder) OR (stress) OR (sleeping disorder) OR (quality of life)) AND (((post covid) OR (long covid) OR (Covid) OR (Sars-cov-2)) AND ((adolescent) OR (child) OR (Juvenile) OR (teenager) OR (youth) OR (young adults) OR (emerging adult)) AND ((Psychology) OR (Psychotherapy) OR (psychiatry)) AND ((online) OR (digital) OR (video-based) OR (tele*)) AND ((effectiveness) OR (efficacy)) AND ((RCT) OR (Randomized controlled trial) OR (Case control) OR (observational cohort))

### Study Selection Process

**Figure 1:**
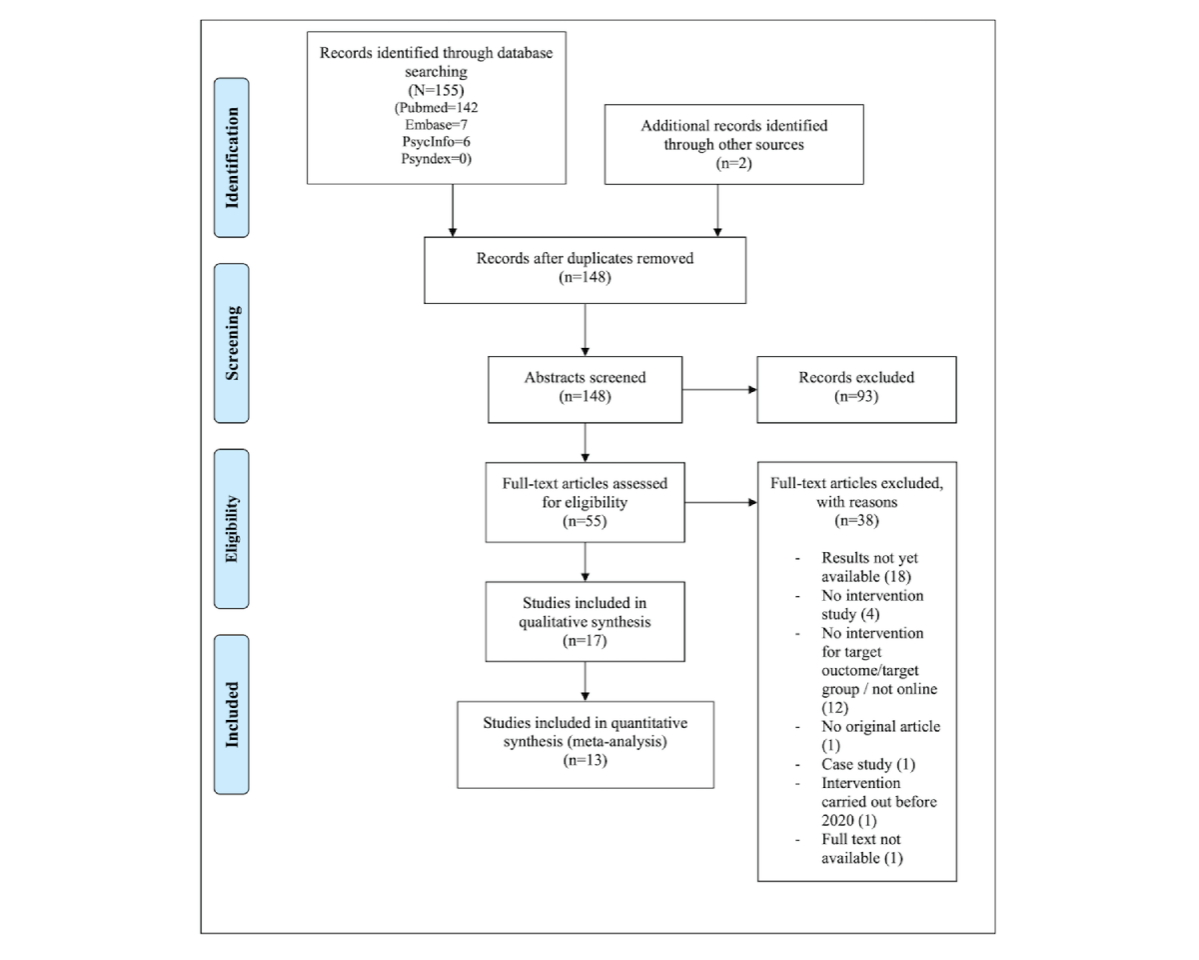
PRISMA (Preferred Reporting Items for Systematic Reviews and Meta-Analysis) flow diagram.

Before examining full texts, 2 authors (LFG, VF) independently screened the titles and abstracts for inclusion and exclusion criteria. In case of a mismatch between the 2 authors, all authors conferred and made a joint decision. See Figure 1 for the detailed exclusion process at each stage. Studies were included if they were (1) original, interventional studies; (2) published not earlier than 2020 (after the onset of the COVID-19 pandemic); (3) in peer-reviewed journals; (4) written in English or German; (5) focused on psychological or psychotherapy interventions that were delivered remotely (eg, online, via mobile app, or via telephone); (6) targeted at mental health issues like distress, depression or anxiety, psychological well-being, or quality of life (QoL); (7) conducted with children, adolescents, or young adults (from the age of 6 years to the age of 30 years, as emerging adulthood is defined as ages up to 30 years [46]). As outcome measures, we included standardized, validated, and reliable instruments designed for children, adolescents, and young adults to assess the listed mental health issues.

### Statistical Analysis

Meta-analyses were conducted to examine the interventions' effectiveness using standardized mean differences (SMDs) as the outcome measure. The SMD compares postintervention scores between treatment and control groups. Only RCTs or pilot RCTs were included in the meta-analyses. A positive SMD indicates lower outcome scores in the treatment group than in the control group. Eligible studies were grouped by outcome type (anxiety, depression, well-being, disordered eating, psychological stress, social functioning, and COVID-19–related outcomes), and separate analyses were carried out for each group. Score polarity had to be reversed in 1 study [47]. Effect sizes were pooled using the “metafor” package [48] in the R environment. A random effects model was fitted to the data to account for variations in sample size, measures, and methodologies between the different studies. Heterogeneity was assessed using Higgins *I*² [49]. Interpretation of the effects sizes is based on Cohen *d* [50,51].

Additionally, a risk of bias assessment for studies included in the meta-analysis was conducted based on the Joanna Briggs Institute Critical Appraisal Checklist for Randomized Controlled Trials and on the Joanna Briggs Institute Critical Appraisal Tool for Quasi-Experimental Studies [52]. All statistical analyses were conducted in the R environment for statistical computing [53].

## Results

### Sample of Included Studies

A total of 155 articles were found in the initial database search process, and 2 additional studies were identified through the reference search strategy. Of the total number of articles, 9 duplicates had to be removed. We examined 55 articles at a full-text level. Of these, 20 articles were excluded since they were not original, were case studies, were not intervention studies, did not target the right outcome variables or groups, were not available, or were carried out before 2020. Additionally, 18 studies had to be excluded at the end of the search process as results still needed to be published for these trials. See Figure 1 for a detailed description of the inclusion and exclusion process.

The final sample of articles in June 2023 comprised 17 articles for the qualitative analysis, with an overall 8732 participants. Of these studies, 13 articles were included in the quantitative analysis. RCTs were reported in 16 articles, whereas 1 study [54] had only a quasiexperimental design with no control group. Only 1 study [55] explicitly compared the online intervention with an intervention conducted in a face-to-face setting. In addition, 1 study [56] was adapted to an online format during data collection due to the beginning of the COVID-19 pandemic.

Of the 17 studies included in the qualitative analysis, 5 (29%) [47,57-60] were conducted in the United States, 4 (24%) [54,61-63] were conducted in China, 2 (12%) each were conducted in Australia [56,64] and the United Kingdom [65,66], and 1 (6%) each was carried out in Canada [67], Italy [55], Iran [19], and Tunisia [68]. Of the included studies, 4 had an approximately (SD 15%) equal distribution of female and male participants [19,54,66,67]. In contrast, 9 had more female participants [47,57-59,61,62,64,65,68], 2 had more male participants [55,63], and 1 study was conducted with female participants only [56]. In addition, 1 additional article reported on 5 studies, of which 4 had more female participants, and 1 had an approximately equal distribution of female and male participants [60]. Of the included articles, 8 focused on children and adolescents [54,55,57-59,62,65,67], while another 8 included young adults [19,47,56,61,63,64,66,68]. The article reporting on 5 studies had samples with only adolescents and samples including young adults [60].

Characteristics of the included studies can be viewed in Tables 1 and 2, and the risk of bias assessment is depicted in Tables 3 and 4.

**Table 1:** Sample characteristics of the included studies.

Study	Sample size	Sample recruitment	Gender	Age	Country
Chen et al (2023) [62]	N=76	Research flyers through social media or school referral	Intervention: female=78.9%; male=21.1%; control: female=76.3%; male=23.7%	Intervention: 11-18 years, mean 16.45 (SD 1.52) years; control: 13-18 years, mean 16.37 (SD 1.24) years	China
Duan et al (2022) [54]	N=76	Online broadcasting platform	Female=56.58%; male=43.42%	10-12 years, mean 10.72 (SD 0.48) years	China
He et al (2022) [63]	N=148	Social media, online platforms, university communities, referred by counselors	Female=37.2%	17-21 years, mean 18.78 (SD 0.89) years	China
Krifa et al (2022) [68]	N=366	Health care students: class visits, posters in university, on website	Female=94%	Mean 20.74 (SD 1.64) years	Tunisia
Kutok et al (2021) [57]	N=80	Targeted Instagram advertisement	Female=59%	13-17 years, mean 15.3 (SD 1.35) years	US
Malboeuf-Hurtubise et al (2021) [67]	N=37	In 2 elementary schools	Female=43%; male=57%	Mean 8.18 years	Canada
Nicol et al (2022) [59]	N=18	Adolescents with depressive symptoms treated in practice-based research networks	Female=88%	13-17 years	US
Pavarini et al (2022) [65]	N=100	Advertisement on social media	Female=84%; male=14%; male (transgender)=1%; nonbinary=1%	16-18 years	UK
Prato et al (2022) [55]	N=40	Patients diagnosed with Tourette syndrome at a child and adolescent neuropsychiatry unit	Female=10%; male=90%	9-16 years, mean 13.5 (SD 2.0) years	Italy
Schleider et al (2022) [58]	N=2452	Instagram advertisements	Female=88.09% (biological sex)	13-16 years	US
Shabahang et al (2021) [19]	N=150	Convenient sample from Guilan University, Iran; online advertisement in college student social network	Female=51.33%; male=48.67%	Mean 24.7 (SD 5.4) years	Iran
Simonsson et al (2021) [66]	N=177	Students from the University of Oxford, UK	Female=64.4%	18-24 years (71.8%)	UK
Suffoletto et al (2021) [47]	N=52 (intervention: n=34; usual care group: n=18)	Young adults with a current mental health diagnosis recruited from primary care or a mental health clinic	Female=85%	Intervention: mean 18.7 (SD 0.42) years; usual care group: mean 18.7 (SD 0.48) years	US
Sun et al (2022) [61]	N=114	University students, online via WeChat-based flyers and websites targeting college students	Female=73.7%	Mean 22.21 (SD 2.67) years	China
Torok et al (2022) [64]	N=455	Social media: persons with suicidal thoughts in the past 12 months	Female=84.4%	18-25 years, mean 21.5 (SD 2.18) years	Australia
Yeager et al (2022) [60]	Study 1: n=2534; study 2: n=790; study 3: n=160; study 4: n=200; study 5: n=119; study 6: n=351	Character Lab Research Network, school students, university students (in specific courses)	Study 1: female=49%, male=49%, nonbinary=2%; study 2: female=64%, male=36%; study 3: female=72.3%, male=27.7%; study 4: female=81.5%, male=18.5%; study 6: similar to study 2	Study 1: 13-18 years; study 2: 17-≥21 years; study 3: 18-26 years; study 4: 18-32 years; study 5: 14-16 years; study 6: similar to study 2	US
Zhou and Wade (2021) [56]	N=100 (pre-COVID-19: n=41; during COVID-19: n=59)	University students at risk of developing an eating disorder	Female=100%	17-26 years; mean 19.85 (SD 2.01) years	Australia

**Table 2:** Study characteristics of the included studies.

Study	Study type	Intervention	Control condition^a^	eHealth technology	Target outcomes	Results
Chen et al (2023) [62]	RCT^b^	Online solution-focused brief therapy (SFBT), active intervention group: 38/76, 50%	No treatment, wait list control, 38/76, 50%	Teleconference	Primary outcome: anxiety; secondary outcomes: depressive symptoms and coping styles	Significant results in intervention group regarding anxiety, depression, and problem-oriented coping styles; depression levels significantly lower in intervention than in control group
Duan et al (2022) [54]	Quasiexperimental, no control group	Online Strength-informed Acceptance and Commitment Therapy (SACT), active intervention group: 76/76, 100%	No control group	Video conferencing system	Quality of life (QoL) and anxiety	Pre to post: significant reduction in anxiety but no significant increase in QoL; pre to 3-month follow-up: reduced anxiety and increased QoL
He et al (2022) [63]	RCT	CBT^c^-based mental health chatbot (XiaoE), active intervention group: 49/148, 33.1%	2 mHealth^d^ minimal active controls: e-book, 49/148, 33.1%; general chatbot: 50/148, 33.8%	Main intervention: XiaoE, unguided CBT-based chatbot, 1 module a day for 1 week	Primary outcome: depressive symptoms; secondary outcomes: working alliance, usability, acceptability	Primary outcome: significant reduction in depression in intervention group compared with e-book and general chatbot group; secondary outcome: better working alliance and acceptability in intervention, no significant difference for usability
Krifa et al (2022) [68]	RCT	CARE^e^ program: internet-based positive psychology intervention, active intervention group: 183/366, 50%	No treatment, wait list control: 183/366, 50%	8-week online self-program: lecture, videos, psychoeducation, practices	Stress, anxiety, depression, emotional regulation, optimism, hope, study engagement, well-being	Significant positive effects in all variables; significant improvement compared with control group
Kutok et al (2021) [57]	RCT	IMPACT^f^, active intervention group: 36/80, 45%	Placebo minimal: enhanced web-based resources: 44/80, 55%	Video intervention plus app-based automated messaging; control: enhanced web-based resources	Cyberbullying: to reduce consequences of cyber victimization, to increase bystander intervention	Feasible, acceptable; improved bystander intervention and well-being in intervention group
Malboeuf-Hurtubise et al (2021) [67]	RCT	MBI^g^ (16/37, 43.2%); P4C^h^ (21/37, 56.8%); both group-based, delivered online	Comparison of 2 active intervention groups (comparative efficacy)	Teleconferencing platform	Anxiety, inattention symptoms, basic psychological need satisfaction (BPN) in the context of COVID-19	P4C: more impact on anxiety and inattention; MBI: better outcomes for BPN
Nicol et al (2022) [59]	Pilot RCT	CBT, active intervention group: 10/18, 55.6%	No treatment, wait list control, 1:1: 8/18, 44.4%	mHealth app with embedded conversational agent	Primary outcomes: depression severity, anxiety; secondary outcomes: feasibility, acceptability, usability	Reduction in symptom severity from moderate to mild in treatment group, no reduction in control group; usability, acceptability, feasibility high
Pavarini et al (2022) [65]	RCT	Online peer support training course “Uplift Peer Support Training,” active intervention group: 50/100, 50%	No treatment, wait list control: 50/100, 50%	Zoom, smaller groups in breakout rooms or via WhatsApp	Primary outcomes: motivation to provide support, perceived support-giving skills, frequency of support provided, compassion toward others, connectedness with peers; secondary outcomes: mental well-being, emotional symptoms, self-efficacy, civic engagement	Primary outcomes: no difference regarding motivation, significant effects of training regarding other primary outcomes; secondary outcomes: significant effect of training
Prato et al (2022) [55]	RCT	Behavior therapy for youths with Tourette syndrome during COVID-19	Non-mHealth evidence-based active control: comparison of online vs face-to-face intervention: 20/40, 50% each	Video conference vs face-to-face	Tic symptoms, obsessive compulsive symptoms, ADHD^i^ symptoms, anxiety, depressive symptoms	Both forms of delivery equally effective regarding most outcomes; online delivery more effective regarding depressive symptoms
Schleider et al (2022) [58]	RCT	Online single-session intervention (SSI) for depressive symptoms (behavioral activation SSI: 821/2452, 33.5% vs growth mindset SSI: 813/2452, 33.2% vs supportive therapy SSI as the control	Placebo active control: supportive control, (structurally similar [eg, matched in length]): 818/2452, 33.4%	Self-administered online intervention	Depressive symptoms, hopelessness, agency, generalized anxiety, COVID-19–related trauma, restrictive eating	Both active SSIs showed significantly better outcomes regarding depression, hopelessness, agency, and restrictive eating than control group; no difference between behavioral action and control group regarding generalized anxiety and COVID-19– related trauma symptoms but between growth mindset and control
Shabahang et al (2021) [19]	RCT	Video-based CBT intervention for COVID-19 anxiety, active intervention group: 75/150, 50%	No treatment, wait list control: 75/150, 50%	Self-administered video-based strategies, online booklet	COVID-19 anxiety, health anxiety, anxiety sensitivity, somatosensory amplification	Significant differences in outcomes between intervention and control groups; high intervention group participant satisfaction with the intervention
Simonsson et al (2021) [66]	RCT	Online, guided, 8-week mindfulness program, active intervention group: 88/177, 50%	No treatment, wait list control: 89/177, 50%	Online classes via Zoom,	Anxiety, depression	Larger reduction in anxiety in treatment group compared with control group; no difference regarding depression
Suffoletto et al (2021) [47]	Pilot RCT	Mobile Support Tool for Mental Health (MoST-MH), active intervention group: 34/52, 65.4%	mHealth minimally active control: enhanced usual care (eUC; weblink to psychoeducational videos): 18/52, 34.6%	Text messaging, web-based check-ins, video feedback (psychoeducation)	Mental health symptoms, mental health self-efficacy, mental health care use	MoST-MH: reduction in all symptoms except substance abuse; eUC: only reduction in general anxiety, family distress, hostility; no improvements regarding self-efficacy and care use in either group
Sun et al (2022) [61]	RCT	Mindfulness-based mHealth intervention, active intervention group: 57/114, 50%	mHealth minimally active control (matched social support mHealth control): 57/114, 50%	Videoconferencing via Zoom, WeChat-based mini-program	Primary outcomes: anxiety, depression; secondary outcomes: mindfulness, social support, emotional suppression	Reduction in anxiety and depression and increase in mindfulness and social support in both groups; greater effect on anxiety through mindfulness intervention; greater engagement with and higher acceptability of mindfulness mHealth
Torok et al (2022) [64]	RCT	Self-guided smartphone app based on DBT^j^, active intervention group: 228/455, 51.1%	Placebo active control, smartphone app with general information: 227/455, 49.9%	Smartphone app (LifeBuoy)	Primary outcome: suicidal ideation symptom severity; secondary outcomes: depression, generalized anxiety, distress, well-being	Significantly higher effects of intervention regarding suicidal ideation; no superior effects regarding secondary outcomes
Yeager et al (2022) [60]	RCT	Synergistic mindset intervention, active intervention group: study 1: 1208/2534, 47.7%; study 2: 387/790, 49%; study 3: 74/160, 46%; study 4: growth only, 52/200, 26%; stress only, 65/200, 32.5%; synergistic, 39/200, 19.5%; study 5: 61/119, 51.3%; study 6: 179/351, 51%	Placebo active control, study 1: 1326/2534, 52.3%; study 2: 403/790, 51.0%; study 3: 86/160, 54%; study 4: 44/200, 22%; study 5: 58/119, 48.7%; study 6: 172/351, 49%	Self-administered online training module	Studies 1 and 2: stress-related cognition; studies 3 and 4: cardiovascular reactivity; studies 4 and 5: psychological well-being; study 5: daily cortisol levels, academic success; study 6: anxiety levels during COVID-19 lockdowns	Improvements in outcomes greater in treatment group than in control group in all experiments
Zhou and Wade (2021) [56]	RCT	Online intervention to reduce disordered eating, active intervention group: 77/100, 77%	No treatment, assessment only control: 23/100, 23%	Online format introduced in April 2021	Disordered eating, body image flexibility, self-compassion, fear of self-compassion, negative affect	Significantly higher symptomology during COVID-19 than pre-COVID-19, active intervention significantly increased self-compassion compared with control, no other significant time x condition effects

^a^Typology of control groups based on Goldberg et al [44].

^b^RCT: randomized controlled trial.

^c^CBT: cognitive behavioral therapy.

^d^mHealth: mobile health.

^e^CARE: Coherence, Attention, Relationship, and Engagement.

^f^IMPACT: Intervention Media to Prevent Adolescent Cyber-Conflict Through Technology.

^g^MBI: mindfulness-based intervention.

^h^P4C: philosophy for children.

^i^ADHD: attention-deficit/hyperactivity disorder.

^j^DBT: dialectical behavior therapy.

**Table 3:** Risk of bias assessment for randomized controlled trials.

First author (year)			True randomization		Concealed allocation	Similar groups at baseline		Participants, personnel, or outcome assessors blinded to assignment		Identical treatment of groups		Follow-up: complete or full description		Analysis of participants in their groups		Outcome measurement: equal and reliable		Appropriate statistical analysis		Appropriate trial design and deviations accounted for
**Randomized controlled trials**																				
	Chen et al (2023) [62]	Yes		Yes		Yes	Participants and personnel: no; outcome assessors: yes		Yes		Yes		Yes		Yes		Yes		Yes	
	He et al (2022) [63]	Yes		Yes		Yes	Yes		Yes		Yes		Yes		Yes		Yes		Yes	
	Krifa et al (2022) [68]	Yes		Unclear		Yes	Unclear		Yes		Yes		Yes		Yes		Yes		Yes	
	Kutok et al (2021) [57]	Randomized but stratified by age and gender		Yes		Yes	Participants and personnel: no; outcome assessors: yes		Yes		Yes		Yes		Yes		Yes		Yes	
	Malboeuf-Hurtubise et al (2021) [67]	Unclear		Unclear		No	Unclear		Yes		No follow-up		Yes		Yes		Yes		No real control group, 2 interventions	
	Nicol et al (2022) [59]	Yes		Yes		Yes	Unclear		Yes		Yes		Yes		Yes		Yes		Yes	
	Pavarini et al (2022) [65]	Yes		Unclear		Yes	Unclear		More assessments in treatment group		Yes		Yes		Yes		Yes		Yes	
	Prato et al (2022) [55]	Yes		Unclear		Yes	Participants and personnel: no; outcome assessors: unclear		Yes		Yes		Yes		Yes		Yes		Yes	
	Schleider et al (2022) [58]	Unclear		Unclear		Yes	Unclear		Yes		Yes		Yes		Yes		Yes		Yes	
	Shabahang et al (2021) [19]	Yes		Unclear		Yes	Unclear		Yes		No follow-up		Yes		Yes		Yes		Yes	
	Simonsson et al, (2021) [66]	Yes		Unclear		Yes	Unclear		Yes		Yes		Yes		Yes		Yes		Yes	
	Suffoletto et al (2021) [47]	Unclear		Yes		Partially	Outcome assessors: yes		Yes		Yes		Yes		Yes		Yes		Yes	
	Sun et al (2022) [61]	Yes		Unclear		Unclear	Participants and research assistant: yes (at first)		Yes		Yes		Yes		Yes		Yes		Yes	
	Torok et al (2022) [64]	Yes		Yes		Yes	Yes		Yes		Yes		Yes		Yes		Yes		Yes	
	Yeager et al (2022) [60]	Yes		Yes		Unclear	Yes		Yes		Unclear		Yes		Yes		Yes		Yes	
	Zhou and Wade (2021) [56]	Yes		Unclear		Yes	Unclear		Yes		Unclear		Yes		Yes		Yes		Sample size intervention and control unbalanced	

**Table 4:** Risk of bias assessment for quasiexperimental studies.

First author (year)			Clear distinction cause and effect		Similar participant in comparison	Similar treatment of comparison group		Control group		Multiple measurement of outcome pre- and postintervention		Follow-up: complete or full description		Equal measurement of participants in comparisons		Reliable measurement of outcomes		Appropriate statistical analysis		
Duan et al (2022) [54]		Yes		Not applicable		Not applicable	No		Unclear		Yes		Not applicable		Yes		Yes			

### Characteristics of Online Interventions Used in the Included Studies

In most (9/17, 53%) of the included studies [54-57,61,62,65-67], different versions of videoconferencing systems were used to deliver the interventions remotely (see Table 2). In an intervention targeted at cyberbullying (Intervention Media to Prevent Adolescent Cyber-Conflict Through Technology [IMPACT]), Kutok et al [57] added app-based automated messaging to their video-delivered intervention. Pavarini et al [65] added the possibility for smaller group discussions by using breakout rooms and WhatsApp for their online peer support training. The mindfulness-based mobile health intervention by Sun et al [61] was supplemented by a WeChat-based mini-program. Other interventions delivered remotely via videoconferencing were the online Strength-informed Acceptance and Commitment Therapy (SACT) [54], mindfulness-based interventions [66,67], philosophy for children (P4C) [67], behavior therapy for Tourette syndrome [55], an intervention to reduce disordered eating [56], and the online solution-focused brief therapy (SFBT), primarily to reduce symptoms of anxiety [62].

Next to these online interventions with teleconferencing systems, 4 studies used self-administered online interventions. Schleider et al [58] examined online single interventions for depressive symptoms, and Shabahang et al [19] targeted COVID-19–related anxiety with self-administered video-based strategies and online booklets. An 8-week self-program with lectures and videos was delivered as an intervention by Krifa et al [68], and Yeager et al [60] used self-administered online training to reduce stress-related symptoms. Text messaging, web-based check-ins, and video feedback with psychoeducation were applied in a study by Suffoletto et al [47] in their Mobile Support Tool for Mental Health (MoST-MH). A cognitive behavioral therapy (CBT)–based mental health chatbot (XiaoE) was used to reduce depressive symptoms by He et al [63]. One study [64] used a smartphone app (LifeBuoy) based on dialectical behavior therapy (DBT) to target suicidal ideation. In contrast, a second study [59] used an app with an embedded conversational agent based on CBT to primarily reduce depressive symptoms.

Not all studies reported on the feasibility and acceptability of their interventions. Those that did, however, found the intervention to be feasible [57,59] and acceptable [57-59,63], met with high satisfaction [19,62], and more accepted and engaging in the treatment group than in the control group [61].

### Effectiveness of Online Interventions Regarding Mental Health Outcomes

Mental health–related outcomes varied in the included studies (see Table 2). They included anxiety, depression, mental well-being, social functioning, COVID-19–related symptoms, cyberbullying, Tourette syndrome, disordered eating, suicidal ideation, and psychological stress, among others.

#### Anxiety

Several studies reported reduced anxiety [19,47,54,55,59-62,66-68]. The impact on anxiety was more prominent in some studies for the treatment group than for the control group [19,59-61,66,68]. In contrast, others found only partial differences [58], equal effects, or no differences between groups [55,64]. P4C had a more significant impact on anxiety than a mindfulness-based intervention in one study [67].

**Figure 2:**
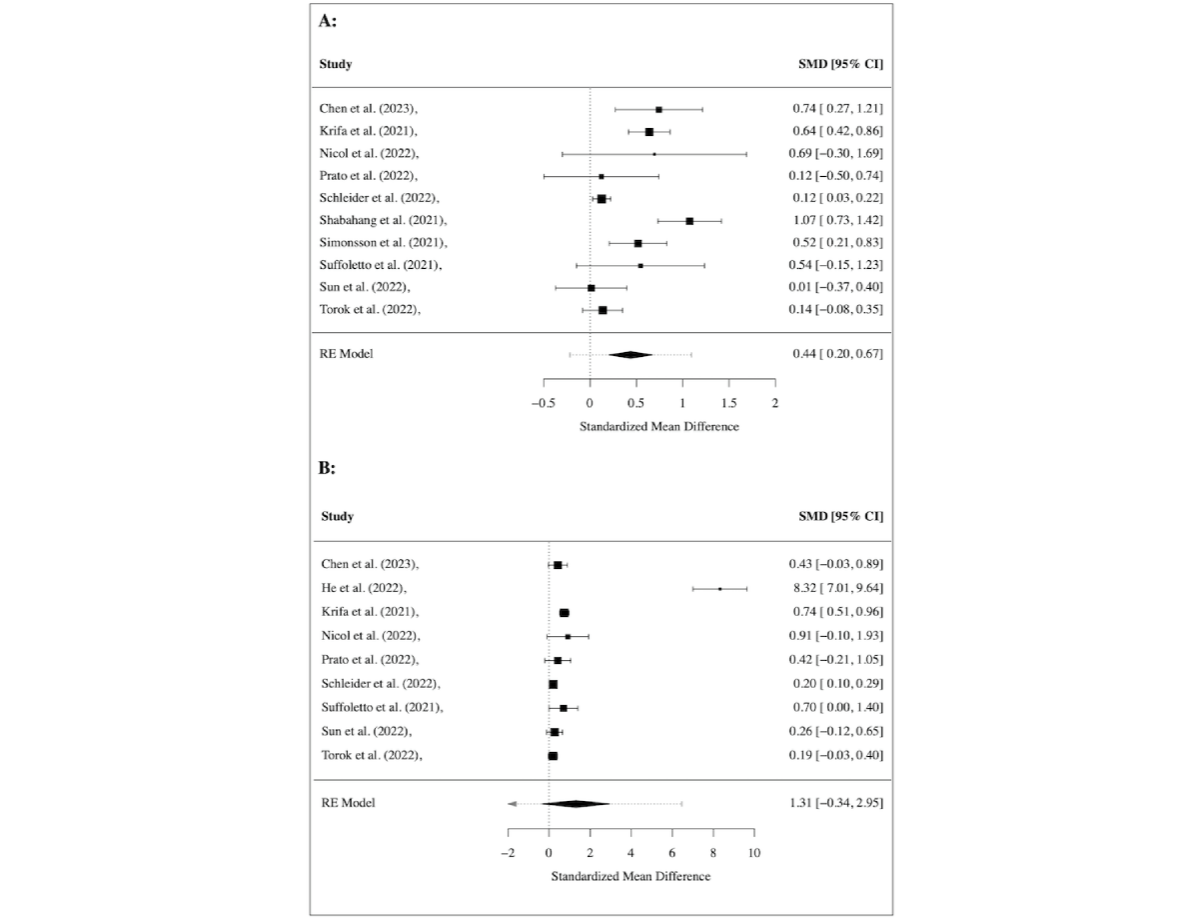
Meta-analysis of treatment effect regarding (A) anxiety and (B) depression, shown using the overall and individual study standardized mean difference (SMD) and 95% CIs (those that include 0 show nonsignificant effects), where a positive effect size indicates a decrease in symptoms.

A meta-analysis of 10 studies, with 9 targeting generalized anxiety disorder and 1 targeting health anxiety, showed an overall significant positive effect of interventions in the form of decreased symptoms (SMD=0.44, 95% CI 0.20 to 0.67; *I*^2^=82.9%). Figure 2A shows a forest plot of the observed outcomes.

#### Depression

Reduced symptoms of depression were found in several studies [47,55,58,59,61-63,68], with superiority of the intervention group found in some [47,55,58,59,62,63,68]. The studies conducted by Sun et al [61], Simonsson et al [66], and Torok et al [64] found no superior effects of the treatment on depressive symptoms.

Nevertheless, a meta-analysis of 9 studies found a strong treatment effect (SMD=1.31, 95% CI 0.34 to 2.95; *I*^2^=99.63%). The observed outcomes are depicted in a forest plot in Figure 2B.

#### Mental Well-Being, Quality of Life, Agency or Self-Efficacy

**Figure 3:**
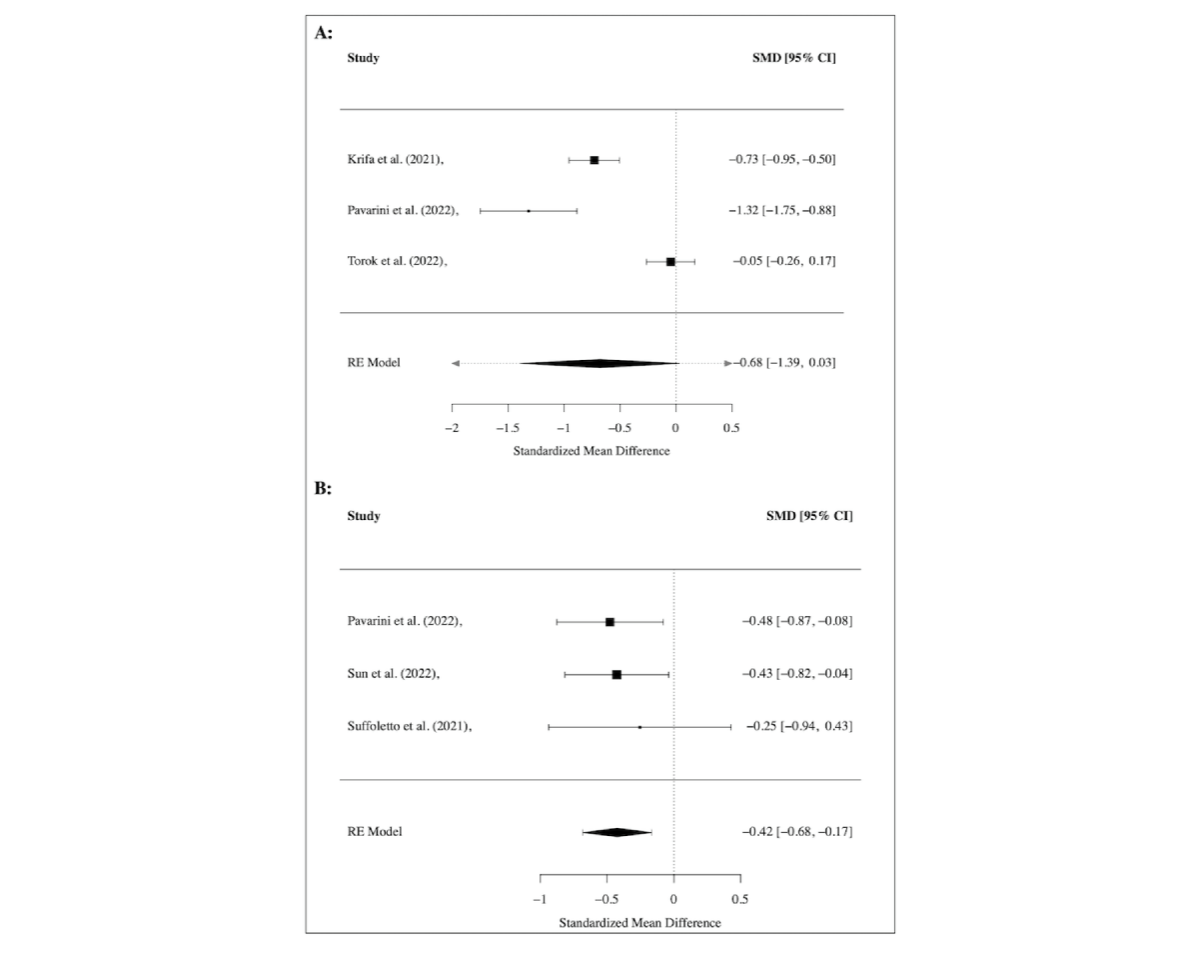
Meta-analysis of treatment effect regarding (A) well-being and (B) social functioning, shown using the overall and individual study standardized mean difference (SMD) and 95% CIs (those that include 0 show nonsignificant effects), where a negative effect size indicates an increase of well-being and social functioning.

Several studies found increased well-being [47,57,60,65,67,68]. QoL was increased at the 3-month follow-up in 1 study [54]. Self-efficacy or agency was increased in some studies [58,65], while 1 study found no effect [47]. Only 3 studies [64,65,68] were eligible for a meta-analysis analyzing the treatment effect on well-being. However, no significant effect was shown in the meta-analysis (see Figure 3A).

#### Other Main Outcomes

A study targeting cyberbullying increased bystander intervention in the treatment group [57], while another showed promising results regarding increased social support-giving skills, compassion toward others, and civic engagement, among other outcomes [65]. Tic and obsessive-compulsive symptoms in children and adolescents with Tourette syndrome were equally reduced via videoconference and face-to-face interventions [55]. Using a smartphone app, 1 study was able to significantly reduce suicidal ideation [64], and 1 study targeting disordered eating increased self-compassion through treatment. At the same time, no other effect was found [56].

**Figure 4:**
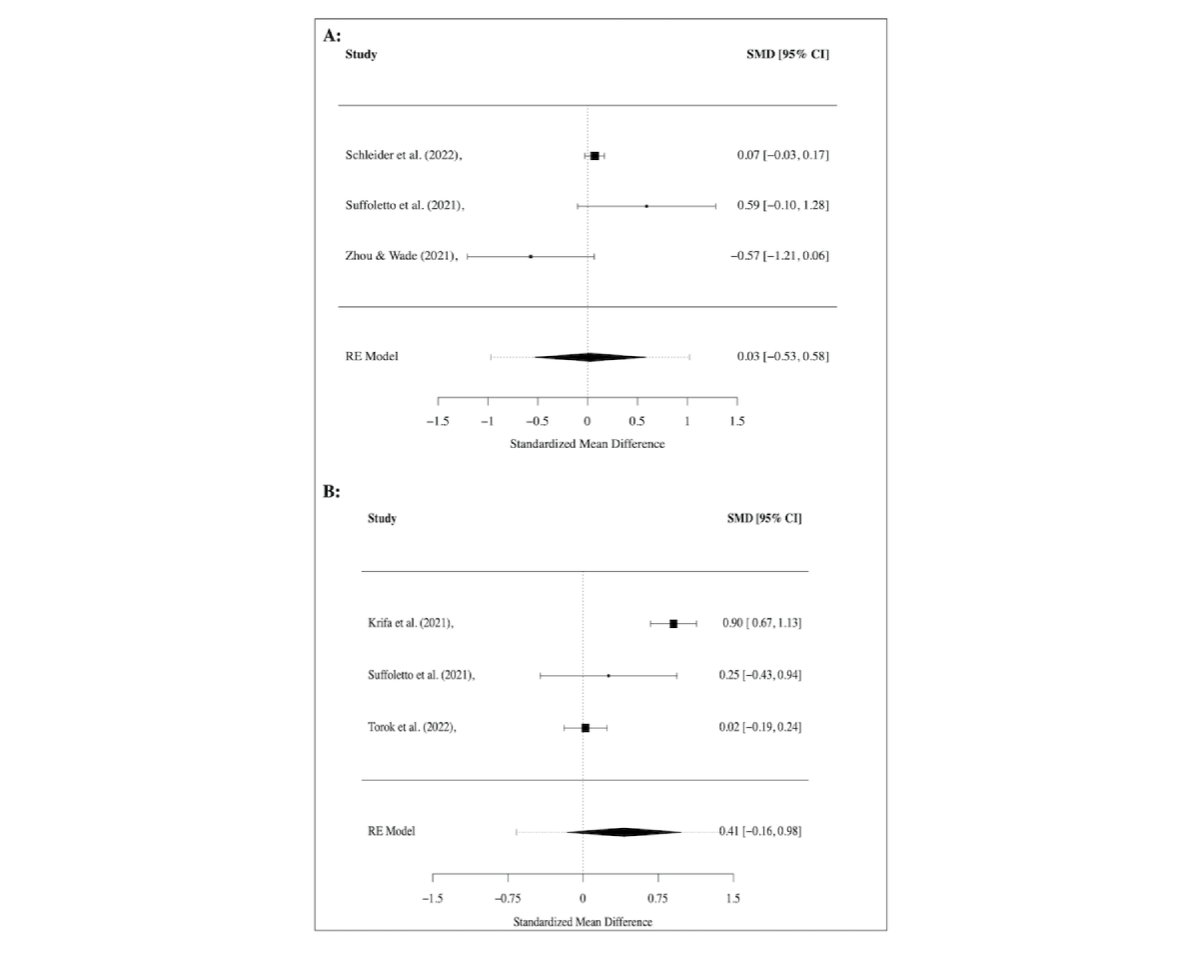
Meta-analysis of treatment effect regarding (A) disordered eating and (B) psychological stress, shown using the overall and individual study standardized mean difference (SMD) and 95% CIs (those that include 0 show nonsignificant effects), where a positive effect size indicates a decrease in symptoms.

**Figure 5:**
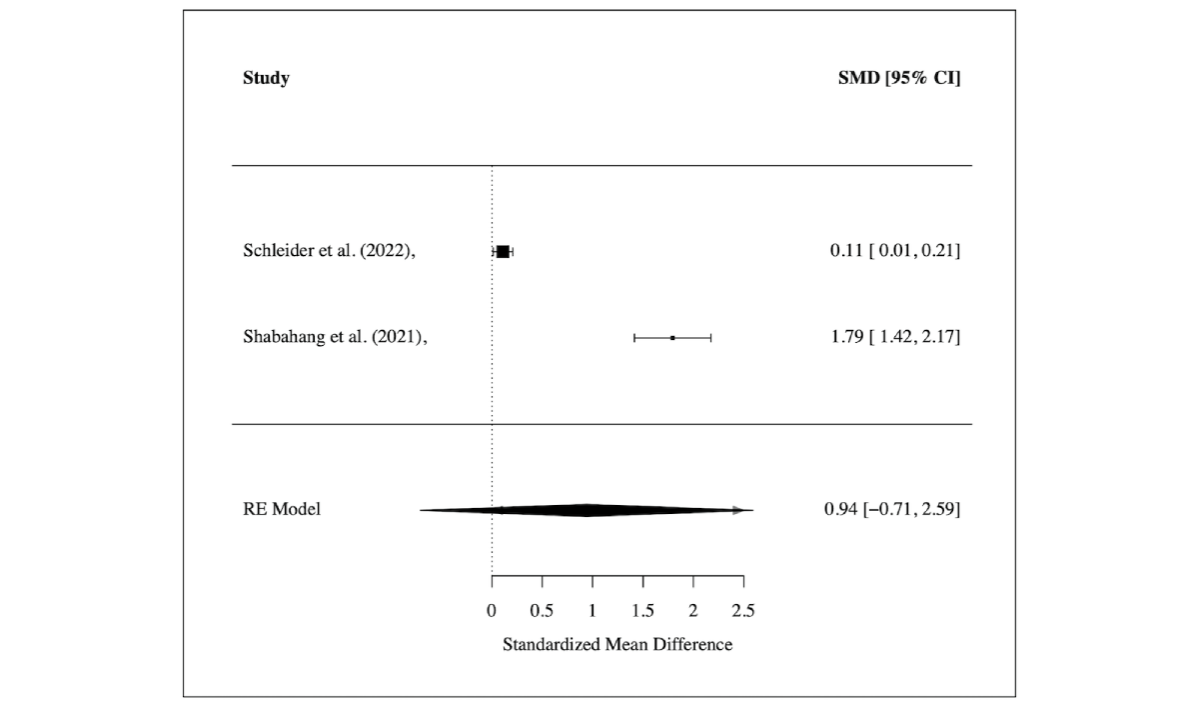
Meta-analysis of treatment effect regarding COVID-19–related symptoms, shown using the overall and individual study standardized mean difference (SMD) and 95% CIs (those that include 0 show nonsignificant effects), where a positive effect size indicates a decrease in symptoms.

Meta-analyses were conducted for several of these outcomes. Regarding disordered eating (see Figure 4A), psychological stress (see Figure 4B), and COVID-19–related trauma or anxiety (see Figure 5), no significant treatment effects were found across studies, while a significant medium effect (SMD=–0.42, 95% CI –0.68 to –0.17; *I*^2^=0.0%) was found for interventions targeting social functioning in 3 studies [47,61,65] (see Figure 3B). For the outcomes of attention and emotional functioning, more data were needed for the meta-analyses.

## Discussion

### Principal Findings

This systematic review and meta-analysis is the first of its kind investigating the effectiveness of online or remote interventions for psychological symptoms and disorders in children, adolescents, and young adults after the onset of the COVID-19 pandemic. We examined 17 studies conducted between the pandemic's start and June 2023 for the impacts of their online interventions. Despite the necessary fast development due to the increased need for remote interventions during the COVID-19 pandemic, the results are promising. All the studies observed positive effects on some of the outcomes they targeted through their remote interventions.

Of the 17 included articles, 16 [19,47,55-68] were RCTs. However, control conditions differed across the RCTs. Only 1 study directly compared an online intervention with a face-to-face intervention [55], which is a control condition that, based on a typology proposed by Goldberg et al [44], provides high comparison strength. The mentioned study found almost equal effects of the online and in-person interventions [55]. Nevertheless, it is expected, due to the great need for fast solutions to deliver interventions amid the ongoing pandemic and obstacles like quarantine, lockdowns, and increased safety measures, that comparisons with face-to-face-interventions were not possible in most cases. Some of the included studies had wait list or no-treatment control groups [19,56,59,62,65,66,68], which can be considered as a control condition with low comparison strength [44]. Others used different content in the control groups [47,57,58,60,61,63,64], mostly providing medium comparison strength [44], or compared different kinds of interventions [58,67] (high comparison strength [44]). These different kinds of control conditions have to be considered when comparing effect sizes of the included studies, and future research should try to use control conditions that provide high comparison strength. Most of the included studies used a videoconferencing system, although several applied interventions that were developed or adapted especially for online delivery.

Meta-analyses on treatment effectiveness yielded significant effects regarding depressive symptoms and medium effects regarding anxiety and social functioning. The results indicate that online or remote interventions show promising results regarding the aforementioned variables. This is a slightly more favorable result than earlier reviews on online interventions or prevention programs with young people conducted before the pandemic. Earlier results were not entirely conclusive, but there were some promising findings for depressive symptoms [69,70] and anxiety [69]. For adults, a review found digitalized CBT interventions to reduce depressive symptoms in pregnant women [41]. However, specifically in the context of COVID-19, a need for more high-quality research has been identified. A systematic review that included only studies with adults published after the onset of the pandemic found some encouraging results for online interventions targeting anxiety and depression [38]. In another review on web-based exercise interventions for adults, the superiority of the interventions over the control conditions was present in only 1 of 3 studies for depressive symptoms and in none for anxiety symptoms [39].

Concerning other variables, the picture is even more unclear: Interventions to improve well-being and reduce psychological stress, disordered eating, and COVID-19–related psychological symptoms did not show significant effects across studies in this meta-analysis. One must consider, however, that only a few studies for each outcome were eligible for these calculations. Only 3 studies could be included in the analyses regarding well-being. One used a Zoom-based intervention focusing on peer support [65], one used an app to reduce suicidal ideation [64], and the third used a self-administered online positive psychology intervention for different mental health outcomes [68]. Thus, in addition to various main interventions using different kinds of online or remote applications, it can be assumed that the baseline state of well-being or differently expressed amount of suffering was quite different between the 3 studies, which might explain the lack of a significant overall treatment effect.

Psychological stress was analyzed in 3 additional studies [47,64,68]; 2 were included in the meta-analysis of interventions for COVID-19–related outcomes [19,58]. There were also 2 different outcomes used in the latter 2 studies: 1 study [58] examined COVID-19–related trauma, while the other [19] focused on COVID-19–related anxiety. However, the systematic review by Bonardi et al [38] found 3 high-quality studies that were able to decrease different COVID-19–related symptoms like anxiety and depressive symptoms in adults postintervention [71] or at least 6 weeks after the intervention [72,73]; this shows that there seems to be some online or remote interventions available for adult participants.

Regarding disordered eating, the lack of overall treatment effect across the studies could indicate that more than remote therapy is needed for eating disorders and symptoms. Eating disorders might require approaches that treat the somatic aspects in a clinical setting to regularly control for treatment compliance [74]. A previous meta-analysis found similar results, with the lowest effectiveness for online interventions for eating disorders [75]. The study by Zhou and Wade [56] compared symptoms before the onset of COVID-19 and during COVID-19 and found more symptoms during the pandemic, underlining the increased need for interventions due to the pandemic. Although disordered eating and body image flexibility decreased in patients entering the study both before and during the pandemic, the impact of the intervention on self-compassion decreased during the pandemic. All in all, for all the variables showing no overall treatment effects, the few studies available suggest that more research is needed before a clear conclusion regarding the effectiveness of remote or online interventions can be drawn for these symptoms.

Most of the included studies based their online interventions primarily on well-studied therapy forms like CBT [19,47,55,57,59,63] and extensions of CBT or therapy forms related to it like acceptance commitment therapy [54], mindfulness-based interventions [61,66,67], and DBT [47,64]. Interventions based on positive psychology [68] and SFBT [62] were also included in the sample. Thus, interventions were developed from evidence-based forms of therapy. No clear superiority of any form of therapy can be found in this sample of studies. Interventions differed according to length, from 1 session [58] to 3 months [47,59]. Nevertheless, even the single-session intervention was effective [58].

Although some studies show promising results regarding interventions for adolescents [55,58-60,62,65] or young adults [19,47,60,61,63,64,66,68], it is more unclear how effective such interventions are for younger children, as only 2 studies focusing on elementary school children [67] or children up to the age of 12 years [54] could be included. In 1 of these studies [67], a philosophy-based intervention was more effective than the mindfulness-based intervention, possibly hinting at a higher effectiveness of more creative approaches when working with younger children.

Most studies recruited through social media, primary care centers, or at a university. However, in 2 articles, schools were involved: 1 study [62] used school referrals, while the other conducted the intervention in elementary school classes [67], thus showing that, especially with younger children, interventions can also be set within the school context, even if online.

It must be noted that most studies were conducted in North America or China. Although it can be assumed that technical opportunities might be equal in most of Europe, it needs to be clarified how the results can be adapted to lower-income countries, where financial aspects might impede technical opportunities.

The digital transition to online or remotely delivered interventions seems essential, not only considering challenging circumstances like the COVID-19 pandemic, which made face-to-face treatment in many cases impossible, but also in light of the ever-increasing numbers of children, adolescents, and young adults experiencing mental health issues or who have psychological disorders. Thus, it is relevant to develop low-threshold interventions [8,9,14,16]. Nevertheless, several factors must be considered: Legal frameworks might need to be adapted for different countries [8], and therapists might need support when transitioning to online interventions [8,27]. Regarding the development of such interventions, studies have shown positive effects by including peer groups in the development process [34,36] and using peers as advisors [35,37].

However, using digital media and smartphones in and of themselves might pose risk factors for children and adolescents: An increase in cyber victimization through media use has been found [57], and young people are more at risk for addictive behavior in general [7]. Problematic behavior has also been discussed for problematic smartphone use [43], which has been found to impede mental well-being and QoL in children and adolescents [76].

The clinical implications of this meta-analysis are both immediate and far-reaching; the results underscore the versatility and applicability of online therapeutic interventions across diverse settings. As the COVID-19 pandemic amplified the demand for remote interventions, the emergence of promising outcomes, despite rapid development, demonstrates the adaptability and resilience of the mental health sector. These findings suggest that online and hybrid therapeutic modalities not only provide a viable alternative to traditional face-to-face sessions but also bridge the accessibility gap. They offer crucial mental health support to those confronted with problems of accessibility rooted in personal, communicative, geographical, or logistical barriers, as well as challenges stemming from limited mobility due to mental or physical disorders. Such restrictions often make traditional therapeutic settings challenging, underscoring the importance of versatile, remote solutions.

The utility of remote interventions and the promising outcomes of these methodologies have transformative potential for various contexts. In educational environments, for instance, schools can leverage online interventions to address the mental health needs of students who may be reluctant or unable to access traditional counseling services [35-37,77]. Primary care settings can also integrate telehealth solutions into their care regimes, ensuring patients have consistent and comprehensive mental health support alongside their physical health needs. Additionally, psychiatric rehabilitation provides supportive care, but the therapeutic effects often decrease after discharge [78]. Implementing online or hybrid care modalities post-inpatient treatment could potentially bolster and prolong the beneficial outcomes of therapy, offering a more sustained therapeutic impact for patients in the long run [79].

Telehealth and hybrid systems can be transformative in delivering mental health services. A hybrid approach, which blends traditional face-to-face therapy with online sessions, can cater to diverse patient needs and preferences, enhancing treatment adherence, accessibility, and comfort. For example, patients might initiate their therapeutic journey via face-to-face consultations and later transition to online sessions for convenience, or vice versa. Schools can adopt similar hybrid models, offering in-person counseling sessions and providing digital platforms for students to access support during out-of-school hours or remote learning periods. Likewise, primary care facilities can offer a combination of in-person consultations with remote follow-ups, ensuring continuity of care. The potential of these strategies will need detailed scientific investigation.

This adaptability was also evident during crises like the Syrian and Ukrainian wars, where online interventions were effectively used to support individuals suffering from trauma and distress in their home countries or while migrating (eg, [80,81]). Therapists and mental health professionals from other countries could remotely provide much-needed assistance, showcasing the potential of such platforms in transcending international borders [82]. The success of these interventions in various crises—health pandemics or geopolitical conflicts—signals the need to reevaluate conventional therapeutic models. A shift toward hybrid care models that combine digital and in-person strategies could be pivotal, especially in future catastrophic events in which immediate physical intervention is hindered [83]. This could ensure that mental health support remains uninterrupted and universally accessible, regardless of geographical and political boundaries.

The universality and adaptability of online interventions suggest a broader shift in the mental health landscape. As the world becomes increasingly interconnected and digital, there is a pressing need to recalibrate therapeutic models. Embracing telehealth and hybrid systems can ensure that mental health support remains robust and adaptable to patients' needs.

### Limitations

Although this meta-analysis and literature review is the first to report the effects of online interventions for children, adolescents, and young adults during the pandemic, some limitations of this systematic review and meta-analysis must be noted. We would like to clarify that only studies with experimental or quasiexperimental designs were included in the systematic review. One of the included studies was not an RCT [54]. However, the results of this study are only reported descriptively, as they were not included in the meta-analysis. As for the type of comparison groups, no restrictions were made due to the novelty of the field, aiming to capture a comprehensive range of experimental approaches. We believe this approach provides a more inclusive representation of the current state of research in this domain. Control groups in most studies do not consist of face-to-face interventions due to the pandemic’s nature, potentially affecting the validity of conclusions about the true effectiveness of online interventions. A risk of bias assessment was carried out for all included studies, revealing, in some cases, a need for more details regarding allocation concealment and blinding of participants, personnel, and outcome assessors. Proper randomization was only evident in certain cases (see Tables 3 and 4), possibly affecting the overall quality of the studies included. Above all, the various examined outcomes and the different media or renditions of the online or remote interventions mean that no 2 studies in the sample looked at the exact same intervention. Although understandable in a rapidly evolving field like online interventions, more consistent research on specific interventions is necessary for in-depth meta-analyses and subanalyses. The sample is relatively small, leading to even smaller sample sizes of the outcome groups that were analyzed quantitatively. When the literature search was conducted, several other studies matching the search criteria were registered, but results were unavailable.

### Conclusion

All in all, the included studies exhibit promising results regarding the implementation of online or app-based interventions for mental health issues for children, adolescents, and young adults. This is relevant not only in times of crises such as the COVID-19 pandemic or catastrophic events but also given the increasing prevalence rates for psychological disorders in these demographics. The results underscore that the digital landscape allows for more straightforward, accessible engagement with young populations, demonstrating equal effectiveness as traditional therapeutic methods. Still, research on this population is limited so far. Notably, online and app-based interventions provide a compelling alternative to face-to-face therapy, showcasing notable efficacy, particularly in addressing symptoms of anxiety and depression and improving social functioning. The commitment from young individuals toward these interventions seems robust and encouraging. Although there is a pressing need for further high-quality research on various interventions with heightened comparability, it is evident that there are tangible, effective alternatives to in-person therapeutic interventions. Considering these findings, incorporating online intervention techniques should be paramount in the future training of clinical psychologists and psychotherapists to ensure they remain adaptive, effective, and relevant in our ever-evolving digital age.

## Appendix

Multimedia Appendix 1 PRISMA (Preferred Reporting Items for Systematic Reviews and Meta-Analysis) checklist.

Multimedia Appendix 2 Database search strategy in more detail.

## Abbreviations

CBT: cognitive behavioral therapy
DBT: dialectical behavior therapy
IMPACT: Intervention Media to Prevent Adolescent Cyber-Conflict Through Technology
MoST-MH: Mobile Support Tool for Mental Health
P4C: philosophy for children
PRISMA: Preferred Reporting Items for Systematic Reviews and Meta-Analysis
QoL: quality of life
RCT: randomized controlled trial
SACT: Strength-informed Acceptance and Commitment Therapy
SFBT: solution-focused brief therapy
SMD: standardized mean difference
