# Supplementary material for: Effectiveness of Online and Remote Interventions for Mental Health in Children, Adolescents, and Young Adults After the Onset of the COVID-19 Pandemic: Systematic Review and Meta-Analysis
Source: JMIR Ment Health. 2024 Feb 5;11:e46637. doi: 10.2196/46637 (PMC10877489; doi:10.2196/46637)
Supplement: Multimedia Appendix 1 [file mental_v11i1e46637_app1.docx]

| **Section and Topic** | **Item #** | **Checklist item** | **Location where item is reported** |
| --- | --- | --- | --- |
| **TITLE** | | |  |
| Title | 1 | Identify the report as a systematic review. | The report is identified as a systematic review in the title (see title). |
| **ABSTRACT** | | |  |
| Abstract | 2 | See the PRISMA 2020 for Abstracts checklist. | The abstract includes the main aspects that are proposed in the PRISMA Abstracts checklist, while at the same time adhering to the journal guidelines for abstracts, e.g. regarding length. For this reason, the risk of bias assessment is not mentioned in the abstract, although we have done a risk of bias assessment.  Regarding funding see the sections acknowledgements and conflict of interest. |
| **INTRODUCTION** | | |  |
| Rationale | 3 | Describe the rationale for the review in the context of existing knowledge. | This has been done in the last sections of the introduction: existing literature as well as systematic reviews that focus on similar topics are mentioned as well as the reason why our research question has not been answered sufficiently before. |
| Objectives | 4 | Provide an explicit statement of the objective(s) or question(s) the review addresses. | This has been done in the last sentence of the introduction section. |
| **METHODS** | | |  |
| Eligibility criteria | 5 | Specify the inclusion and exclusion criteria for the review and how studies were grouped for the syntheses. | The inclusion and exclusion criteria are mentioned in the paragraph “study selection process”.  In the section “statistical analyses” we describe which studies were included in the meta-analysis and how they were grouped for these calculations (I.e., by outcome type). |
| Information sources | 6 | Specify all databases, registers, websites, organisations, reference lists and other sources searched or consulted to identify studies. Specify the date when each source was last searched or consulted. | The databases etc. are listed in the section “search strategy” as well as in textbox 1. In the section “search strategy” we also mention the last date of retrieval (June 2023). |
| Search strategy | 7 | Present the full search strategies for all databases, registers and websites, including any filters and limits used. | This is described in textbox 1. |
| Selection process | 8 | Specify the methods used to decide whether a study met the inclusion criteria of the review, including how many reviewers screened each record and each report retrieved, whether they worked independently, and if applicable, details of automation tools used in the process. | This is described in the section “study selection process”. |
| Data collection process | 9 | Specify the methods used to collect data from reports, including how many reviewers collected data from each report, whether they worked independently, any processes for obtaining or confirming data from study investigators, and if applicable, details of automation tools used in the process. | Information on this can be found in the section “study selection process”. |
| Data items | 10a | List and define all outcomes for which data were sought. Specify whether all results that were compatible with each outcome domain in each study were sought (e.g. for all measures, time points, analyses), and if not, the methods used to decide which results to collect. | The outcomes are listed in the section “study selection process” as well as in textbox 1. It is also stated in this section that only studies with validated, standardized measures were included. |
|  | 10b | List and define all other variables for which data were sought (e.g. participant and intervention characteristics, funding sources). Describe any assumptions made about any missing or unclear information. | Other variables which were defined for the search like patient characteristics (e.g., age groups), and intervention characteristics (e.g., online, via app, …) are mentioned in the section “study selection process” as well as in textbox 1. |
| Study risk of bias assessment | 11 | Specify the methods used to assess risk of bias in the included studies, including details of the tool(s) used, how many reviewers assessed each study and whether they worked independently, and if applicable, details of automation tools used in the process. | The method for the risk of bias assessment is mentioned in the section “statistical analysis”. |
| Effect measures | 12 | Specify for each outcome the effect measure(s) (e.g. risk ratio, mean difference) used in the synthesis or presentation of results. | Information on this (i.e., standardized mean difference, interpretation of effect sizes) can be found in the section “statistical analysis). |
| Synthesis methods | 13a | Describe the processes used to decide which studies were eligible for each synthesis (e.g. tabulating the study intervention characteristics and comparing against the planned groups for each synthesis (item #5)). | This is described in the section “statistical analysis”. |
|  | 13b | Describe any methods required to prepare the data for presentation or synthesis, such as handling of missing summary statistics, or data conversions. | This is described in the section “statistical analysis” (e.g. reversion of score polarity in one study). |
|  | 13c | Describe any methods used to tabulate or visually display results of individual studies and syntheses. | Sample and study characteristics are described in table 1 and 2, for the meta-analysis data on standardized mean differences will be shown in individual plots for each outcome. |
|  | 13d | Describe any methods used to synthesize results and provide a rationale for the choice(s). If meta-analysis was performed, describe the model(s), method(s) to identify the presence and extent of statistical heterogeneity, and software package(s) used. | This is described in the section “statistical analysis”. |
|  | 13e | Describe any methods used to explore possible causes of heterogeneity among study results (e.g. subgroup analysis, meta-regression). | This is described in the section “statistical analysis” (i.e., fitting of random effects model, assessment of heterogeneity with Higgins *I*²). |
|  | 13f | Describe any sensitivity analyses conducted to assess robustness of the synthesized results. | No sensitivity analyses were conducted to determine the robustness of the review's findings as the number of studies were too low to conduct sensitivity analyses. |
| Reporting bias assessment | 14 | Describe any methods used to assess risk of bias due to missing results in a synthesis (arising from reporting biases). | It is described in the “statistical analysis” section which criteria were applied for inclusion of studies in the meta-analysis. In the results section for each outcome it is described which studies could in fact be included. In cases of studies where outcomes were not reported, authors were contacted to request additional data or information on said outcomes. |
| Certainty assessment | 15 | Describe any methods used to assess certainty (or confidence) in the body of evidence for an outcome. | To assess certainty, the authors reached a consensus on the confidence level of the evidence for each study, aiming to identify and exclude those with severe flaws or a high risk of bias that compromised the evidence's integrity. |
| **RESULTS** | | |  |
| Study selection | 16a | Describe the results of the search and selection process, from the number of records identified in the search to the number of studies included in the review, ideally using a flow diagram. | This is described in the section “sample of included studies”. Figure 1 (flow chart) also gives an overview of the inclusion /exclusion process. |
|  | 16b | Cite studies that might appear to meet the inclusion criteria, but which were excluded, and explain why they were excluded. | Reasons for exclusion after full screen are described in the section “sample of included studies”. |
| Study characteristics | 17 | Cite each included study and present its characteristics. | This is also done in the section “sample of included studies”, e.g. designs of the studies, country of origin and gender distribution are mentioned.  Table 1 also gives an extensive overview of sample characteristics. |
| Risk of bias in studies | 18 | Present assessments of risk of bias for each included study. | This is done in table 3. |
| Results of individual studies | 19 | For all outcomes, present, for each study: (a) summary statistics for each group (where appropriate) and (b) an effect estimate and its precision (e.g. confidence/credible interval), ideally using structured tables or plots. | Individual results on outcomes are presented in table 2. Summaries across studies for outcomes are given in the individual outcome sections in the result section.  Individual plots are presented for each outcome showing standardized mean differences of all included studies. |
| Results of syntheses | 20a | For each synthesis, briefly summarise the characteristics and risk of bias among contributing studies. | Characteristics for each outcome are summed up in the individual outcome sections. |
|  | 20b | Present results of all statistical syntheses conducted. If meta-analysis was done, present for each the summary estimate and its precision (e.g. confidence/credible interval) and measures of statistical heterogeneity. If comparing groups, describe the direction of the effect. | The results of the meta-analysis are described in the in the individual outcome sections, reporting standardized mean differences, confidence intervals, measure of heterogeneity, and direction of effect. |
|  | 20c | Present results of all investigations of possible causes of heterogeneity among study results. | Differences between studies reporting on the same outcomes are described in the individual outcome sections as well as in the section of characteristics. |
|  | 20d | Present results of all sensitivity analyses conducted to assess the robustness of the synthesized results. | See Item #13f, no sensitivity analyses were conducted. |
| Reporting biases | 21 | Present assessments of risk of bias due to missing results (arising from reporting biases) for each synthesis assessed. | See item #14, no risk of bias assessment was done due to exclusion of studies based on other criteria than missing data. However, possible implications are discussed in the “limitations” section of the discussion. |
| Certainty of evidence | 22 | Present assessments of certainty (or confidence) in the body of evidence for each outcome assessed. | Based on the assessement of certainty we did not exclude any data set based on the evidence for each outcome assessed. |
| **DISCUSSION** | | |  |
| Discussion | 23a | Provide a general interpretation of the results in the context of other evidence. | This is done extensively in the “principal findings” section of the discussion. |
|  | 23b | Discuss any limitations of the evidence included in the review. | This is described in the “limitations” section of the article. |
|  | 23c | Discuss any limitations of the review processes used. | This is described in the “limitations” section of the article. |
|  | 23d | Discuss implications of the results for practice, policy, and future research. | Some of this is discussed at the end of the “principal findings” section of the discussion, while implications for future research are additionally mentioned in the “limitations” section, and, again, a summary of implications for practice, policy, and future research is given in the “conclusions” section. |
| **OTHER INFORMATION** | | |  |
| Registration and protocol | 24a | Provide registration information for the review, including register name and registration number, or state that the review was not registered. | This review was not registered. |
|  | 24b | Indicate where the review protocol can be accessed, or state that a protocol was not prepared. | The review was not registered and no protocol can be retrieved online, but information on the procedure can be obtained from the authors. |
|  | 24c | Describe and explain any amendments to information provided at registration or in the protocol. | Since the review was not registered, this is not applicable. |
| Support | 25 | Describe sources of financial or non-financial support for the review, and the role of the funders or sponsors in the review. | The NÖ Landesgesundheitsagentur, legal entity of University Hospitals in Lower Austria, provided the organizational framework to conduct this research. We also want to acknowledge support from the Open Access Publishing Fund of Karl Landsteiner University of Health Sciences, Krems, Austria. See section “acknowledgements" in the paper. |
| Competing interests | 26 | Declare any competing interests of review authors. | No competing interests of review authors are declared. See section “conflicts of interests”. |
| Availability of data, code and other materials | 27 | Report which of the following are publicly available and where they can be found: template data collection forms; data extracted from included studies; data used for all analyses; analytic code; any other materials used in the review. | The search strategy is supplied in an appendix to the publication. Other data like the analytic code can be obtained from the authors. |

*From:*  Page MJ, McKenzie JE, Bossuyt PM, Boutron I, Hoffmann TC, Mulrow CD, et al. The PRISMA 2020 statement: an updated guideline for reporting systematic reviews. BMJ 2021;372:n71. doi: 10.1136/bmj.n71

For more information, visit: <http://www.prisma-statement.org/>
