# Supplementary material for: Effectiveness of Online and Remote Interventions for Mental Health in Children, Adolescents, and Young Adults After the Onset of the COVID-19 Pandemic: Systematic Review and Meta-Analysis
Source: JMIR Ment Health. 2024 Feb 5;11:e46637. doi: 10.2196/46637 (PMC10877489; doi:10.2196/46637)
Supplement: Multimedia Appendix 2 [file mental_v11i1e46637_app2.docx]

# Multimedia Appendix 2

## Databases searched:

1. PubMed
2. PsycInfo
3. Psyndex
4. Embase
5. Google Scholar

Search strategy: combine searches of psychopathology, COVID-19, sample age characteristics, psychological/psychiatric interventions and digital interventions

## Complete Search Term

(((depression) OR (anxiety) OR (mental health) OR (eating disorder) OR (stress) OR (sleeping disorder) OR (quality of life)) AND (((post covid) OR (long covid) OR (Covid) OR (Sars-cov-2)) AND ((adolescent) OR (child) OR (Juvenile) OR (teenager) OR (youth) OR (young adults) OR (emerging adult)) AND ((Psychology) OR (Psychotherapy) OR (psychiatry)) AND ((online) OR (digital) OR (video-based) OR (tele*)) AND ((effectiveness) OR (efficacy)) AND ((RCT) OR (Randomized controlled trial) OR (Case control) OR (observational cohort))

## Psychopathology terms:

1. Depression
2. Anxiety
3. Mental health
4. Eating disorder
5. Stress
6. Sleeping disorder
7. Quality of live

## COVID-19 terms:

1. Post covid
2. Long covid
3. Covid
4. Sars-cov-2

## Sample age characteristic terms:

1. Adolescent
2. Child
3. Juvenile
4. Teenager
5. Youth
6. Young adults
7. Emerging adults

## Psychological/psychiatric intervention terms:

1. Psychology
2. Psychotherapy
3. Psychiatry

## Digital intervention terms:

1. Online
2. Digital
3. Video-based
4. Tele*

## Databases were also searched for specific research methods:

1. RCT
2. Randomized controlled trial
3. Case control
4. Observational cohort

## Other terms:

1. Effectiveness
2. Efficacy
